# Editorial: Community series in gut feelings: investigating the link between microbiota and kidney disease progression, volume II

**DOI:** 10.3389/fimmu.2026.1915260

**Published:** 2026-09-03

**Authors:** Marijn M. Speeckaert, Joris R. Delanghe

**Affiliations:** 1 Department of Nephrology, Ghent University Hospital, Ghent, Belgium; 2 Research Foundation Flanders (FWO), Brussels, Belgium; 3 Department of Diagnostic Sciences, Ghent University, Ghent, Belgium

**Keywords:** chronic kidney disease, gut microbiota, gut-kidney axis, intestinal dysbiosis, microbial metabolites, microbiome-targeted therapies

Over the past decade, the gut-kidney axis has evolved from a theoretical framework to the primary model for explaining the development and progression of chronic kidney disease (CKD). Fundamental studies show that the intestinal microbiota of CKD patients is markedly altered, and researchers have recognized the central role of gut metabolites in driving inflammation, cardiovascular disease, and further kidney damage (1–5). These findings have fundamentally changed how we view the microbiome, no longer as a spectator but as an active player in disease onset. At the same time, advances in next-generation sequencing, metabolomics, and systems biology have revealed that changes in microbial composition and metabolism are not merely consequences of deteriorating kidney function but also key drivers of metabolism, immune regulation, and organ-to-organ communication (4–6). As a result, the microbiome has become a valuable source of new biomarkers, therapeutic targets, and precision medicine strategies in nephrology.

One of the main points highlighted by prior papers is that microbiome research is increasingly moving beyond describing the microbiota in the body to determining exactly how the microbes living in our gut affect the progression of kidney diseases. Initially, researchers were primarily interested in identifying which types of bacteria were present in patients versus in healthy people. Today, however, the role of gut microbial imbalance and its effects on the host receives more attention. For example, examined changes in the gut microbiota in patients with IgA nephropathy (IgAN) according to the Oxford classification. They compared the gut microbes of patients with and without segmental glomerulosclerosis, a major predictor of poor kidney function. Not only did patients with segmental sclerosis show increased abundance of bacterial taxa and metabolic pathways associated with inflammatory responses and secondary bile acid metabolism, but patients without sclerosis also showed microbial functions involved in cellular homeostasis and detoxification. This may indicate that changes in the microbiome could contribute not only to the general deterioration of disease but also to the formation of specific structural kidney lesions.

review thoroughly explains the mechanistic basis of such findings. It offers a timely, integrated update on connections among intestinal dysbiosis, microbial metabolites, inflammation, and kidney damage. It identifies the toxic effects of renal toxins, including indoxyl sulfate, p-cresyl sulfate, and trimethylamine-N-oxide. These are linked to oxidative stress, endothelial dysfunction, fibrosis, cardiovascular diseases, and the gradual loss of renal function (2, 7, 8). This review also discusses beneficial microbial metabolites, mainly short-chain fatty acids, which help maintain epithelial barrier integrity, modulate immune responses, and exert anti-inflammatory and renoprotective effects (4, 5). By integrating mechanistic, translational, and clinical data, the review positions microbial metabolism as a key interface between the intestinal environment and renal function.

The idea of turning microbiome science into a practical tool has been discussed in more detail by in their review. They critically evaluate existing methods for altering gut bacteria to treat CKD. The approaches mentioned include dietary modulation, prebiotics, probiotics, synbiotics, fecal microbiota transplantation (FMT), and phage therapy, a relatively novel concept. Although many of these methods have shown promising biological results, there remains a lack of clear evidence demonstrating changes in the most important clinical outcomes. This difficulty is part of a broader challenge in the field. Although the link between dysbiosis and CKD is now indisputably recognized, proving a cause-and-effect relationship and translating a better understanding of mechanisms into new, successful treatments remain among the main objectives. The review highlights the need for well-executed randomized controlled trials with uniform outcome measures and long-term studies to determine whether interventions targeting the microbiome have a significant impact on the disease course.

A further key highlight of this Research Topic is the recognition that microbiome-targeted mechanisms are not limited to the gradual deterioration of kidney function. In their study, explore the role of gut bacteria in chronic kidney disease-mineral and bone disorder (CKD-MBD), thereby introducing the concept of a gut-kidney-bone axis. Their review describes how alterations in the gut microbiota induce chronic low-grade inflammation, impair the intestinal barrier, increase uremic toxin levels, and disrupt the endocrine pathways of fibroblast growth factor 23, Klotho, and parathyroid hormone. These interlinked processes account for mineral metabolism defects and bone disorders, two major issues in the later stages of CKD. This evidence further supports the idea that the microbiome is a systemic regulator that affects multiple organ systems through interlinked inflammatory, metabolic, endocrine, and immunological mechanisms.

Collectively, the papers of this Research Topic demonstrate how microbiome research in nephrology has been transformed. Not only can researchers now identify bacterial species, but the field can also uncover functional pathways, measure microbial metabolites, identify microbial disease signatures, and even design targeted interventions. In addition, the studies highlight that kidney disease should not be considered solely a disorder of the organ. On the contrary, CKD is a systemic illness that results from the complex crosstalk among the gut microbiota, the immune system, metabolism, environmental exposures, and remote organs. The fusion of microbiome science with genomics, transcriptomics, metabolomics, and sophisticated computational methods can revolutionize the field by enabling the discovery of new biomarkers, improving risk prediction, and guiding the development of individualized therapies.

Still, significant obstacles remain in this area. Future scientific investigations should move from simple associations to causal inference in microbial dysbiosis and its impact on renal injury and clinical outcomes. To increase reproducibility and enable cross-study comparisons, it would be critical to standardize microbiome sampling, sequencing, and bioinformatic pipelines, as well as reporting practices. Disease progression and treatment response could be more reliably tracked using longitudinal cohorts that combine multi-omics technologies with thorough clinical phenotyping. Research into therapies that affect the microbiome should also include clinical interventions to demonstrate clear improvements in renal, cardiovascular, and patient-centered outcomes. Not only must we overcome these obstacles, but doing so will also become the basis for moving the microbiome from experimental potential to clinical reality.

## References

[1] Vaziri ND Wong J Pahl M Piceno YM Yuan J DeSantis TZ . Chronic kidney disease alters intestinal microbial flora. Kidney Int. (2013) 83:308–15. doi: 10.1038/ki.2012.345 22992469

[2] Meijers BKI Evenepoel P . The gut-kidney axis: indoxyl sulfate, p-cresyl sulfate and CKD progression. Nephrol Dialysis Transplant. (2011) 26:759–61. doi: 10.1093/ndt/gfq818 21343587

[3] Vaziri ND Zhao Y-Y Pahl MV . Altered intestinal microbial flora and impaired epithelial barrier structure and function in CKD: the nature, mechanisms, consequences and potential treatment. Nephrol Dial Transplant. (2016) 31:737–46. doi: 10.1093/ndt/gfv095 25883197

[4] Anders H-J Andersen K Stecher B . The intestinal microbiota, a leaky gut, and abnormal immunity in kidney disease. Kidney Int. (2013) 83:1010–6. doi: 10.1038/ki.2012.440 23325079

[5] Hobby GP Karaduta O Dusio GF Singh M Zybailov BL Arthur JM . Chronic kidney disease and the gut microbiome. Am J Physiol Renal Physiol. (2019) 316:F1211–7. doi: 10.1152/ajprenal.00298.2018 30864840 PMC6620595

[6] Ramezani A Raj DS . The gut microbiome, kidney disease, and targeted interventions. J Am Soc Nephrol. (2014) 25:657–70. doi: 10.1681/ASN.2013080905 24231662 PMC3968507

[7] Barreto FC Barreto DV Liabeuf S Meert N Glorieux G Temmar M . Serum indoxyl sulfate is associated with vascular disease and mortality in chronic kidney disease patients. Clin J Am Soc Nephrol. (2009) 4:1551–8. doi: 10.2215/CJN.03980609 19696217 PMC2758258

[8] Tang WHW Wang Z Kennedy DJ Wu Y Buffa JA Agatisa-Boyle B . Gut microbiota-dependent trimethylamine *N* -oxide (TMAO) pathway contributes to both development of renal insufficiency and mortality risk in chronic kidney disease. Circ Res. (2015) 116:448–55. doi: 10.1161/CIRCRESAHA.116.305360 25599331 PMC4312512

